# Four novel species of *Distoseptispora* (*Distoseptisporaceae*, *Distoseptisporales*) revealed by morphology and phylogenetic analyses from southern China

**DOI:** 10.3897/mycokeys.140.207506

**Published:** 2026-09-08

**Authors:** Fang-Hua Guo, Zhi-Hao Wang, Ming-Gen Liao, Lian-Hu Zhang, Ru-Qiang Cui, Zhao-Huan Xu, Wei-Gang Kuang, Jian Ma

**Affiliations:** 1 College of Agronomy, Jiangxi Agricultural University, Nanchang, Jiangxi 330045, China College of Agronomy, Jiangxi Agricultural University Nanchang China; 2 Key Laboratory of Pest Monitoring and Control for Horticultural Crops, Nanchang, Jiangxi 330045, China Key Laboratory of Pest Monitoring and Control for Horticultural Crops Nanchang China; 3 Jiangxi Key Laboratory for Excavation and Utilization of Agricultural Microorganisms, Jiangxi Agricultural University, Nanchang, Jiangxi 330045, China Jiangxi Key Laboratory for Excavation and Utilization of Agricultural Microorganisms, Jiangxi Agricultural University Nanchang China

**Keywords:** *
Ascomycota
*, molecular phylogeny, new species, *sporidesmium*-like fungi, taxonomy

## Abstract

During investigations of saprobic fungi on decaying wood in terrestrial habitats of southern China, several *sporidesmium*-like isolates were collected from dead branches of unidentified plants in Fujian, Jiangxi, and Yunnan provinces. Based on morphological comparisons and multilocus phylogenetic analyses of ITS, LSU, *RPB2*, and *TEF1* sequence data, four new species of *Distoseptispora*, namely *D. acropleurogena*, *D. distoseptata*, *D. ellipsoidea*, and *D. jiulianshanensis*, are proposed. Detailed descriptions and comparisons with allied taxa are provided. This study enriches the species diversity of *Distoseptispora* in southern China and highlights the importance of integrating morphological and molecular data for species delimitation.

## Introduction

*Sporidesmium*-like fungi are highly diverse and widely distributed and have been commonly recorded from rotten wood, dead branches, and decaying leaves in terrestrial and freshwater habitats (Ellis 1958, 1971, 1976; Wu and Zhuang 2005; Index Fungorum 2026). These fungi show distinct morphological features and are easily characterized by holoblastic phragmoconidia produced on proliferating or non-proliferating conidiophores (Subramanian 1992; Wu and Zhuang 2005). These fungi, which comprise *Sporidesmiumsensu lato* and several segregate or related genera such as *Ellisembia* Subram., *Linkosia* A. Hern.-Gut. & B. Sutton, *Neosporidesmium* Mercado & J. Mena, *Repetophragma* Subram., *Sporidesmiella* P.M. Kirk, and *Stanjehughesia* Subram. (Wu and Zhuang 2005; Seifert et al. 2011), belong to *Dothideomycetes* O.E. Erikss. & Winka and *Sordariomycetes* O.E. Erikss. & Winka in phylogenetic studies (Wijayawardene et al. 2020). Traditional taxonomy of *sporidesmium*-like fungi mainly depends on the presence or absence of conidiophores, the type of conidiophore proliferation, and conidial morphology. However, with advances in phylogenetic analysis, the traditional classification system based on morphological characteristics is increasingly controversial, and more research on *sporidesmium*-like fungi is needed.

*Distoseptispora* K.D. Hyde, McKenzie & Maharachch. is a *sporidesmium*-like genus in the family *Distoseptisporaceae* K.D. Hyde & McKenzie of the order *Distoseptisporales* Z.L. Luo, K.D. Hyde & Hong Y. Su within *Sordariomycetes* (Luo et al. 2019). The genus was established by Su et al. (2016) based on multilocus phylogenies together with morphology and typified by *D. fluminicola* McKenzie, Hong Y. Su, Z.L. Luo & K.D. Hyde. The genus was mainly characterized by solitary, acrogenous, distoseptate conidia seceding schizolytically from integrated, terminal, monoblastic conidiogenous cells on distinct, determinate or percurrently extending conidiophores (Su et al. 2016). Subsequently, the generic concept has been broadened to encompass unusual species with euseptate conidia (e.g., Yang et al. 2018; Karimi et al. 2024; Liao et al. 2025) and even polyblastic conidiogenous cells (e.g., Hyde et al. 2019; Karimi et al. 2024; Liao et al. 2025). Most species of *Distoseptispora* produce distoseptate or euseptate conidia, but one species, *D. martinii* (J.L. Crane & Dumont) J.W. Xia & X.G. Zhang, with muriform conidia, is known (Xia et al. 2017). To date, 130 species have been listed in the genus (Index Fungorum 2026), with the majority reported from southern China and Thailand, highlighting these areas as diversity hotspots for the genus. Liu et al. (2023) provided a checklist of 63 *Distoseptispora* species (61 asexual and two sexual) reported worldwide, including details for each species based on records in the related published literature. Since then, 66 additional species have been described in the genus (Index Fungorum 2026), 24 of which are presented in Liao et al. (2025) following the same format as Liu et al. (2023). However, *D. submersa* Z.L. Luo, K.D. Hyde & H.Y. Su was determined to be a synonym of *D. tectonae* Doilom & K.D. Hyde by Dong et al. (2021) based on minor nucleotide differences and high morphological similarity. “*Distoseptispora keviniligustrina*” H.Z. Du & Jian K. Liu and “*D. guanxiensis*” D.F. Bao & X.G. Tian (Art. 40.5: the holotype was cited as conserved in more than one specimen) were not validly published based on the rules of the International Code of Nomenclature for Algae, Fungi, and Plants (Madrid Code) (Turland et al. 2025). Thus, *Distoseptispora* currently includes 127 valid taxa, all of which were identified based on morphological and phylogenetic analyses.

China spans a wide range of climatic zones, from cold-temperate to tropical. The southern region, characterized by high annual precipitation and warm temperatures, provides favorable conditions for fungal growth and multiplication, resulting in the discovery of more fungal species (Wang and Cai 2023). During ongoing investigations of saprobic fungi in southern China, several species with distinctive *sporidesmium*-like morphological features were isolated from dead branches of unidentified plants in Fujian, Jiangxi, and Yunnan provinces. Multilocus (ITS, LSU, *RPB2*, and *TEF1*) phylogenetic analyses reveal their taxonomic placement within the genus *Distoseptispora*. Based on morpho-phylogenetic evidence, four new species of *Distoseptispora*, namely *D. acropleurogena*, *D. distoseptata*, *D. ellipsoidea*, and *D. jiulianshanensis*, are introduced. Their detailed descriptions and comparisons with allied taxa are presented. This study contributes to the understanding of the species diversity and geographic distribution of *Distoseptispora* in southern China.

## Materials and methods

### Sample collection, fungal isolation, and morphological observation

Samples were collected from forest ecosystems in southern China, primarily from dead branches of unidentified broadleaf trees in terrestrial habitats. The samples were placed in sealed plastic bags with collection details (Rathnayaka et al. 2025) and transported to the laboratory. Specimen processing followed the methods described by Ma et al. (2011). Fungal colonies on the surface of dead branches were examined under a stereomicroscope (Motic SMZ-168, Xiamen, China) at magnifications ranging from 0.75× to 5×. Small amounts of mycelium or conidia from target colonies were picked using sterile needles, transferred to glass slides, and mounted in a drop of lactic acid-phenol solution (lactic acid: phenol: glycerol: sterile water = 1 : 1 : 2 : 1). After covering with a coverslip, the microscopic morphological characteristics were examined and recorded on slides using an Olympus BX 53 light microscope equipped with an Olympus DP 27 digital camera (Olympus Optical Co., Ltd., Tokyo, Japan). For each key structure (e.g., conidiophores and conidia), more than 30 units were measured. Morphological parameters, including length, width (at the broadest part), and septum number, were recorded. All measurements are given as a range (minimum–maximum), with the mean and number (*n*) indicated in parentheses. Length and width are separated by “×.”

Pure cultures were obtained using a single-spore isolation method. A sterile toothpick dipped in sterile water was used to collect individual conidia from the surface of dead branches under 5× magnification. The conidia were transferred to potato dextrose agar (PDA: potato 200 g/L, glucose 20 g/L, agar 20 g/L) plates and incubated overnight at 25 °C in the dark. After conidial germination, single germinated conidia were transferred to fresh PDA plates and incubated at 25 °C in the dark to obtain pure cultures. Colony morphology was observed at regular intervals, and the colony diameter, color, texture, margin characteristics, and pigment production were recorded. The purified strains were preserved in 10% sterile glycerin at 4 °C. All voucher specimens and living cultures were deposited in the Herbarium of Jiangxi Agricultural University, Plant Pathology, Nanchang, China (HJAUP). Taxonomic information on the new taxa was registered in Index Fungorum (Index Fungorum 2026).

### DNA extraction, PCR amplification, and sequencing

The purified strains were incubated on PDA plates at 25 °C in the dark for 4–5 weeks. Surface mycelia were scraped off, and total genomic DNA was extracted using a Fungal Genomic DNA Extraction Kit (Beijing Solarbio Science & Technology Co., Ltd., Beijing, China). All operations were performed strictly in accordance with the manufacturer’s instructions. Four gene loci (ITS, LSU, *RPB2*, and *TEF1*) were chosen for amplification in this study. The primer pairs and PCR conditions used are listed below: ITS: ITS5/ITS4 (White et al. 1990); LSU: 28S1-F/28S3-R (Xia et al. 2017); *RPB2*: RPB2-5F2 (Sung et al. 2007)/fRPB2-7cR (Liu et al. 1999); and *TEF1*: EF1-983F/EF1-2218R (Rehner and Buckley 2005; Zhao et al. 2018). The total volume of each PCR reaction was 20 μL, containing 10 μL of 2× Power Taq PCR MasterMix, 0.8 μL of each forward and reverse primer, 1 μL of DNA template, and 7.4 μL of double-distilled water. The PCR thermal profile was programmed as follows: initial denaturation at 94 °C for 3 min; 35 cycles of denaturation at 94 °C for 15 s, annealing at 54 °C (for ITS, LSU, and *TEF1*) or 59 °C (for *RPB2*) for 15 s, and extension at 72 °C for 30 s; and a final extension at 72 °C for 5 min. The amplified products were detected by 1% agarose gel electrophoresis using StarStain Red Enhanced Nucleic Acid (Beijing Qihengxing Biotechnology Co., Ltd., Beijing, China) and subsequently sent to Hunan Youkanglai Biotechnology Co., Ltd. for bidirectional sequencing. The obtained sequences have been submitted to the GenBank database (www.ncbi.nlm.nih.gov, accessed on 26 July 2026; Table 1).

**Table 1.** List of *Distoseptispora* species and GenBank accession numbers used in the phylogenetic analyses.

**Taxon**	**Strain number**	**GenBank accession numbers**			
		**ITS**	**LSU**	***RPB2***	***TEF1***
*Aquapteridospora aquatica*	MFLUCC 17-2371^T^	MW286493	MW287767	–	–
*A. fusiformis*	MFLUCC 18-1601^T^	MK828652	MK849798	–	MN194056
*A. lignicola*	MFLUCC 15-0377^T^	MZ868774	KU221018	MZ892986	MZ892980
*Cancellidium applanatum*	CBS 337.76^T^	MH860985	MH872755	–	–
*C. cinereum*	MFLUCC 18-0424^T^	MT370353	MT370363	MT370486	MT370488
*Fluminicola saprophytica*	MFLUCC 15-0976^T^	MF374358	MF374367	MF370954	MF370956
*Myrmecridium banksiae*	CBS 132536^T^	JX069871	JX069855	–	–
*M. schulzeri*	CBS 100.54	EU041769	EU041826	–	–
***Distoseptispora acropleurogena***	**HJAUP C2537^T^**	**PZ201931**	**PZ201932**	**PZ583092**	**PZ661937**
***D. acropleurogena***	**HJAUP C2537.2**	**PZ543650**	**PZ546037**	**PZ583093**	**PZ583100**
*D. adscendens*	HKUCC 10820	–	DQ408561	DQ435092	–
*D. amniculi*	MFLUCC 17-2129**^T^**	MZ868770	MZ868761	MZ892982	–
*D. appendiculata*	MFLUCC 18-0259^T^	MN163009	MN163023	–	MN174866
*D. aqualignicola*	KUNCC 21-10729^T^	OK341186	ON400845	OP413474	OP413480
*D. aquamyces*	KUNCC 21-10732^T^	OK341187	OK341199	OP413476	OP413482
*D. aquatica*	S-965	MK828647	MK849792	MN124537	MN194051
*D. aquisubtropica*	GZCC 22-0075^T^	ON527933	ON527941	ON533685	ON533677
*D. arecacearum*	MFLUCC 23-0212	OR354399	OR510860	OR481048	OR481045
*D. atroviridis*	GZCC 20-0511^T^	MZ868772	MZ868763	MZ892984	MZ892978
*D. bambusae*	MFLUCC 20-0091^T^	MT232713	MT232718	MT232881	MT232880
*D. bambusicola*	GZCC 21-0667^T^	MZ474873	MZ474872	–	OM272845
*D. bangkokensis*	MFLUCC 18-0262^T^	MZ518205	MZ518206	–	OK067246
*D. bawanglingensis*	SAUCC WZS13‐1^T^	PQ799295	PQ804721	PQ849357	PQ849363
*D. bawanglingensis*	SAUCC WZS13‐2	PQ799296	PQ804722	PQ849358	PQ849364
*D. cangshanensis*	MFLUCC 16-0970^T^	MG979754	MG979761	–	MG988419
*D. caricis*	CPC 36498^T^	MN562124	MN567632	MN556805	–
*D. changjiangensis*	SAUCC WZS14‐1^T^	PQ799297	PQ804723	PQ849359	PQ849366
*D. chengduensis*	CGMCC 3.27439^T^	PQ067913	PQ067744	–	PQ278565
*D. chiangraiensis*	MFLU 21-0105^T^	MZ890145	MZ890139	–	MZ892970
*D. chinensis*	GZCC 21-0665^T^	MZ474871	MZ474867	–	MZ501609
*D. chishuiensis*	GZCC 23-0729^T^	PP584670	PP584767	–	PP663310
*D. clematidis*	MFLUCC 17-2145^T^	MT310661	MT214617	MT394721	–
*D. combreticola*	CGMCC 3.27721^T^	PQ189782	PQ184737	–	–
*D. crassispora*	KUMCC 21-10726 ^T^	OK310698	OK341196	OP413473	OP413479
*D. curvularia*	KUMCC 21-10725^T^	OK310697	OK341195	OP413472	OP413478
*D. cylindricospora*	DLUCC 1906^T^	OK491122	OK513523	–	OK524220
*D. daanyuanensis*	SAUCC 12326‐1^T^	PV670056	PV670405	–	PV708057
*D. davidalangii*	UESTCC 24.0236^T^	PQ189781	PQ184736	PQ380000	PQ346519
*D. davidii*	GZCC 24-0091^T^	PV820360	PV856177	PZ366469	PZ366443
*D. dehongensis*	KUMCC 18-0090^T^	MK085061	MK079662	–	MK087659
*D. dinghuensis*	ZHKUCC 23-0958^T^	PQ037957	PQ037956	PQ035180	PQ035181
*D. dipterocarpi*	MFLUCC 22-0104^T^	OP600053	OP600052	OP595140	–
*D. dujuanhuensis*	KUNCC 23-13772^T^	PQ845849	PV536297	PX233767	PX238245
***D. distoseptata***	**HJAUP C2360.1^T^**	**PZ560005**	**PZ560000**	**PZ583088**	**PZ583096**
***D. distoseptata***	**HJAUP C2360.2**	**PZ560006**	**PZ560002**	**PZ583089**	**PZ583097**
*D. effusa*	GZCC 19-0532^T^	MW133916	MZ227224	–	MZ206156
*D. eleiodoxae*	MFLUCC 23-0214	OR354398	OR510859	OR481047	OR481044
***D. ellipsoidea***	**HJAUP C1199^T^**	**PZ153136**	**PZ153153**	**PZ180002**	**PZ180007**
***D. ellipsoidea***	**HJAUP C1199.2**	**PZ543651**	**PZ546038**	**PZ583091**	**PZ583099**
*D. euseptata*	MFLUCC 20-0154^T^	MW081539	MW081544	MW151860	–
*D. fasciculata*	KUMCC 19-0081^T^	MW286501	MW287775	–	MW396656
*D. fluminicola*	DLUCC 0391	MG979755	MG979762	–	MG988420
*D. fujianensis*	HJAUP C2509^T^	PQ211095	PQ211103	PQ303679	PQ303682
*D. fusiformis*	GZCC 20-0512^T^	MZ868773	MZ868764	MZ892985	MZ892979
*D. ganzhouensis*	HJAUP C1090^T^	PQ211100	PQ211108	–	PQ303687
*D. gasaensis*	HJAUP C2034^T^	OQ942896	OQ942891	–	OQ944455
*D. gelatinosa*	MFLU 24-0292	PQ570855	PQ570872	–	–
*D. guanshanensis*	HJAUP C1063^T^	OQ942894	OQ942898	OQ944458	OQ944452
*D. guanxiensis*	GZCC 26-0130^T^	PX889843	PX984884	–	–
*D. guanxiensis*	GZCC 26-0131	PX984883	PX984885	PZ013919	PZ013918
*D. guizhouensis*	GZCC 21-0666^T^	MZ474868	MZ474869	MZ501611	MZ501610
*D. guttulata*	MFLU 17-0852^T^	MF077543	MF077554	–	MF135651
*D. hainanensis*	GZCC 22-2047^T^	OR427328	OR438894	OR449119	OR449122
*D. heptapleuricola*	CGMCC 3.27740^T^	PQ189783	PQ184738	PQ380001	PQ346520
*D. hongheensis*	KUNCC 23-14299^T^	PQ845851	PV536299	PX233769	PX238247
*D. hyalina*	MFLUCC 17-2128^T^	MZ868769	MZ868760	MZ892981	MZ892976
*D. hydei*	MFLUCC 20-0481^T^	MT734661	MT742830	MT767128	–
*D. jianfenglingensis*	SAUCC WZS65‐3^T^	PQ799299	PQ804725	PQ849361	PQ849367
*D. jianfenglingensis*	SAUCC WZS65‐4	PQ799300	PQ804726	PQ849362	PQ849368
*D. jingdongensis*	KUNCC 23-13382^T^	PQ845852	–	PX233770	PX238248
*D. jinghongensis*	HJAUP C2120^T^	OQ942897	OQ942893	–	OQ944456
***D. jiulianshanensis***	**HJAUP C1147.1^T^**	**PZ560003**	**PZ559999**	**PZ583086**	**PZ583094**
***D. jiulianshanensis***	**HJAUP C1147.2**	**PZ560004**	**PZ560001**	**PZ583087**	**PZ583095**
*D. keviniana*	GZCC 24-0289^T^	PV820358	–	PZ366468	PZ366441
*D. keviniligustrina*	CGMCC 3.27722^T^	PQ191052	PQ184727	PQ379955	PQ346501
*D. lancangjiangensis*	DLUCC 1864^T^	MW723055	MW879522	–	–
*D. lanceolatispora*	GZCC 22-2045^T^	OR427329	OR438895	OR449120	OR449123
*D. leonensis*	HKUCC 10822	–	DQ408566	DQ435089	–
*D. licualae*	MFLUCC 14-1163A^T^	ON650686	ON650675	–	ON734007
*D. lignicola*	MFLUCC 18-0198^T^	MK828651	MK849797	–	–
*D. liupanshuiensis*	GZCC 23-0730^T^	PP584669	PP584766	-	PP663309
*D. longispora*	HFJAU 0705^T^	MH555359	MH555357	–	–
*D. longissima*	GMBC5340^T^	PV932969	PV932988	PX373357	PX392331
*D. longnanensis*	HJAUP C1040^T^	OQ942887	OQ942886	–	OQ944451
*D. mangrovei*	ZHKUCC 25-0793	PX806213	PX811045	PX965873	PX965874
*D. martinii*	CGMCC 3.18651^T^	KU999975	KX033566	–	–
*D. meilingensis*	JAUCC 4727^T^	OK562390	OK562396	–	OK562408
*D. menghaiensis*	HJAUP C2045^T^	OQ942890	OQ942900	–	–
*D. menglunensis*	HJAUP C2170^T^	OQ942899	OQ942888	OQ944461	OQ944457
*D. mengsongensis*	HJAUP C2126^T^	OP787876	OP787874	–	OP961937
*D. monospora*	HKAS：145690	PQ898696	PQ898699	PV001672	PV001669
*D. motuoensis*	KUNCC 24-17628^T^	PP600327	PP621731	–	PP639546
*D. muchuanensis*	CGMCC 3.27444^T^	PQ067919	PQ067750	–	PQ278571
*D. multiseptata*	MFLUCC 15-0609^T^	KX710145	KX710140	–	MF135659
*D. nabanheensis*	HJAUP C2003^T^	OP787873	OP787877	–	OP961935
*D. nanchangensis*	HJAUP C1074^T^	OQ942889	OQ942895	OQ944460	OQ944454
*D. nanpingensis*	HJAUP C2517^T^	PQ211096	PQ211104	PQ303678	–
*D. narathiwatensis*	MFLUCC 23-0215^T^	OR234708	OR510858	OR250443	OR250440
*D. neorostrata*	MFLUCC 18-0376^T^	MN163008	MN163017	–	–
*D. nonrostrata*	KUNCC 21-10730^T^	OK310699	OK341198	OP413475	OP413481
*D. obclavata*	MFLUCC 18-0329^T^	MN163012	MN163010	–	–
*D. obpyriformis*	MFLUCC 17-1694^T^	–	MG979764	MG988415	MG988422
*D. olivaceoviridis*	MFLU 24-0290	PQ568144	PQ569325	–	–
*D. pachyconidia*	KUMCC 21-10724^T^	OK310696	OK341194	OP413471	OP413477
*D. palmarum*	MFLUCC 18-1446^T^	MK085062	MK079663	MK087670	MK087660
*D. phangngaensis*	MFLUCC 16-0857^T^	MF077545	MF077556	–	MF135653
*D. phragmiticola*	GUCC 220201^T^	OP749887	OP749880	OP752699	OP749891
*D. polyblasta*	GZCC 25-0526^T^	PV820359	PV856176	–	PZ366442
*D. pseudoaquisubtropica*	GZCC 24-0061	PV820361	PV856178	–	PZ366444
*D. pulchra*	KUNCC 23-17029^T^	PQ469943	PQ576433	PQ587326	PQ587327
*D. quinqueseptata*	GMBC5341^T^	PV932966	PV932985	PX373358	PX392328
*D. rayongensis*	MFLUCC 18-0415^T^	MH457172	MH457137	MH463255	MH463253
*D. rostrata*	MFLUCC 16-0969^T^	MG979758	MG979766	MG988417	MG988424
*D. saprophytica*	MFLUCC 18-1238^T^	MW286506	MW287780	MW504069	MW396651
*D. septata*	GZCC 22-0078^T^	ON527939	ON527947	ON533690	ON533683
*D. sichuanensis*	KUNCC 23-15519^T^	PP584672	PP584769	–	PP663312
*D. sinensis*	HJAUP C2044^T^	OP787878	OP787875	–	OP961936
*D. solitaria*	GZCC 24-0038^T^	PV820362	PV856179	–	–
*D. songkhlaensis*	MFLUCC 18-1234^T^	MW286482	MW287755	–	MW396642
*D. suae*	CGMCC 3.24262^T^	OQ874968	OQ732679	OQ870341	OR367670
*D. submersa*	MFLUCC 16-0946	MG979760	MG979768	MG988418	MG988426
*D. subtropica*	HJAUP C2528^T^	PQ211099	PQ211107	PQ303677	PQ303684
*D. suoluoensis*	MFLUCC 17-0224^T^	MF077546	MF077557	–	MF135654
*D. tectonae*	MFLUCC 12-0291^T^	KX751711	KX751713	KX751708	KX751710
*D. tectonigena*	MFLUCC 12-0292^T^	KX751712	KX751714	KX751709	–
*D. terrestris*	HJAUP C2539^T^	PV448667	PV450538	–	PV469764
*D. thailandica*	MFLUCC 16-0270^T^	MH275060	MH260292	–	MH412767
*D. thysanolaenae*	KUN-HKAS：102247^T^	MK045851	MK064091	–	MK086031
*D. tongrensis*	GMBC5343^T^	PV932970	PV932989	PX373360	–
*D. tropica*	GZCC 22-0076^T^	ON527935	ON527943	ON533687	ON533679
*D. uncariicola*	UESTCC 24.0229	PQ189784	PQ184739	PQ380002	PQ346521
*D. vaginae*	GZCC 25-0527^T^	PV820363	PV856180	–	PZ366445
*D. velvetica*	GZCC 24-0286^T^	PV820364	PV856181	–	PZ366446
*D. verrucosa*	GZCC 20-0434^T^	MZ868771	MZ868762	MZ892983	MZ892977
*D. wuyishanensis*	HJAUP C2515^T^	PV448666	PV450537	PV469759	PV469763
*D. wuzhishanensis*	GZCC 22-0077^T^	ON527938	ON527946	–	ON533682
*D. xingpingensis*	KUNCC 22-12667^T^	OQ874970	OQ732681	OQ870340	OR367671
*D. xishuangbannaensis*	KUMCC 17-0290^T^	MH275061	MH260293	MH412754	MH412768
*D. yichunensis*	HJAUP C1065^T^	OQ942885	OQ942892	OQ944459	OQ944453
*D. yongxiuensis*	JAUCC 4725^T^	OK562388	OK562394	–	OK562406
*D. yunjushanensis*	JAUCC 4723^T^	OK562392	OK562398	–	OK562410
*D. yunnansis*	MFLUCC 20-0153^T^	MW081541	MW081546	MW151861	MW084995
*D. zhejiangensis*	HJAUP C2588^T^	PV448668	PV450539	–	PV469765
*D. zunyiensis*	GZCC 23-0734^T^	PQ248941	–	PQ614176	PQ605053

Notes: The ex-type cultures are indicated by “T” after strain numbers; “–” stands for no sequence data in GenBank. **CBS**: Centraalbureau voor Schimmelcultures, Westerdijk Fungal Biodiversity Institute, Utrecht, The Netherlands; **CGMCC**: China General Microbiological Culture Collection Center, Institute of Microbiology, Chinese Academy of Sciences, Beijing, China; **CPC**: Collection of P.W. Crous, Westerdijk Fungal Biodiversity Institute, Utrecht, The Netherlands; **DLUCC**: Dali University Culture Collection, Yunnan, China; **GMBC**: Guangxi Microbiology Biotechnology Culture Collection, Guangxi, China; **GUCC**: Guangxi University Culture Collection, Guangxi, China; **GZCC**: Guizhou Culture Collection, Guizhou, China; **HFJAU**: Herbarium of Fungi, Jiangxi Agricultural University, Jiangxi, China; **HJAUP**: Jiangxi Agricultural University Plant Pathology Culture Collection, Jiangxi, China; **HKAS**: Herbarium of Cryptogams, Kunming Institute of Botany, Chinese Academy of Sciences, Yunnan, China; **HKUCC**: The University of Hong Kong Culture Collection, Hong Kong, China; **JAUCC**: Jiangxi Agricultural University Culture Collection, Jiangxi, China; **KUMCC**: Kunming University of Science and Technology Culture Collection, Yunnan, China; **KUNCC**: Kunming Institute of Botany Culture Collection, Chinese Academy of Sciences, Yunnan, China; **MFLU**: Herbarium of Mae Fah Luang University, Chiang Rai, Thailand; **MFLUCC**: Mae Fah Luang University Culture Collection, Chiang Rai, Thailand; **SAUCC**: Hainan Academy of Agricultural Sciences Culture Collection, Hainan, China; **UESTCC**: University of Electronic Science and Technology of China Culture Collection, Sichuan, China; **ZHKUCC**: Zhongkai University of Agriculture and Engineering Culture Collection, Guangdong, China.

### Phylogenetic analyses

Reference sequences of closely related species of *Distoseptispora* were downloaded from GenBank and combined with newly generated sequences from this study (Table 1). Sequence alignments for each locus (ITS, LSU, *RPB2*, and *TEF1*) were performed separately using the online version of MAFFT v.7 (https://mafft.cbrc.jp/alignment/server/, Katoh et al. 2019). Alignments were then manually refined and trimmed using MEGA v.7 (Kumar et al. 2016) to remove ambiguous regions and ensure uniform sequence boundaries across taxa. The four trimmed datasets were subsequently concatenated into a single matrix using the “Concatenate Sequence” function in PhyloSuite v1.2.2 (Zhang et al. 2020).

Maximum-likelihood (ML) analyses were performed using IQ-TREE (Nguyen et al. 2015) with an edge-linked partition model. ModelFinder (Kalyaanamoorthy et al. 2017) was used to select the best-fit nucleotide substitution model for each alignment dataset based on the Bayesian information criterion (BIC). In ML phylogenies, the best-fit model was TIM2e+I+G4 for ITS, TIM2+F+R4 for LSU and *TEF1*, and GTR+F+R4 for *RPB2*. A total of 10,000 ultrafast bootstrap replicates were performed to evaluate branch support (Minh et al. 2013). Bayesian inference (BI) analyses were conducted in MrBayes 3.2.6 (Ronquist et al. 2012) under a partitioned model. Two independent chains were run for 2,000,000 generations, and sampling was performed every 1,000 generations. The first 25% of sampled trees were discarded as the burn-in phase. The optimal substitution models for each partition—SYM+I+G4 for ITS and SYM+I+G4 for LSU, *RPB2*, and *TEF1*—were selected according to the corrected Akaike information criterion (AICc). The reconstructed phylogenetic trees were visualized with FigTree v1.4.4 and further typeset and polished using Adobe Illustrator CS5. Bootstrap values (BS ≥ 75%) from ML analyses and posterior probabilities (PP ≥ 0.90) from BI analyses were annotated at the corresponding nodes. *Myrmecridium banksiae* (CBS 132536) and *M. schulzeri* (CBS 100.54) were selected as outgroup taxa. The sequences generated in this study were deposited in GenBank.

## Results

### Molecular phylogeny

**Figure 1.**
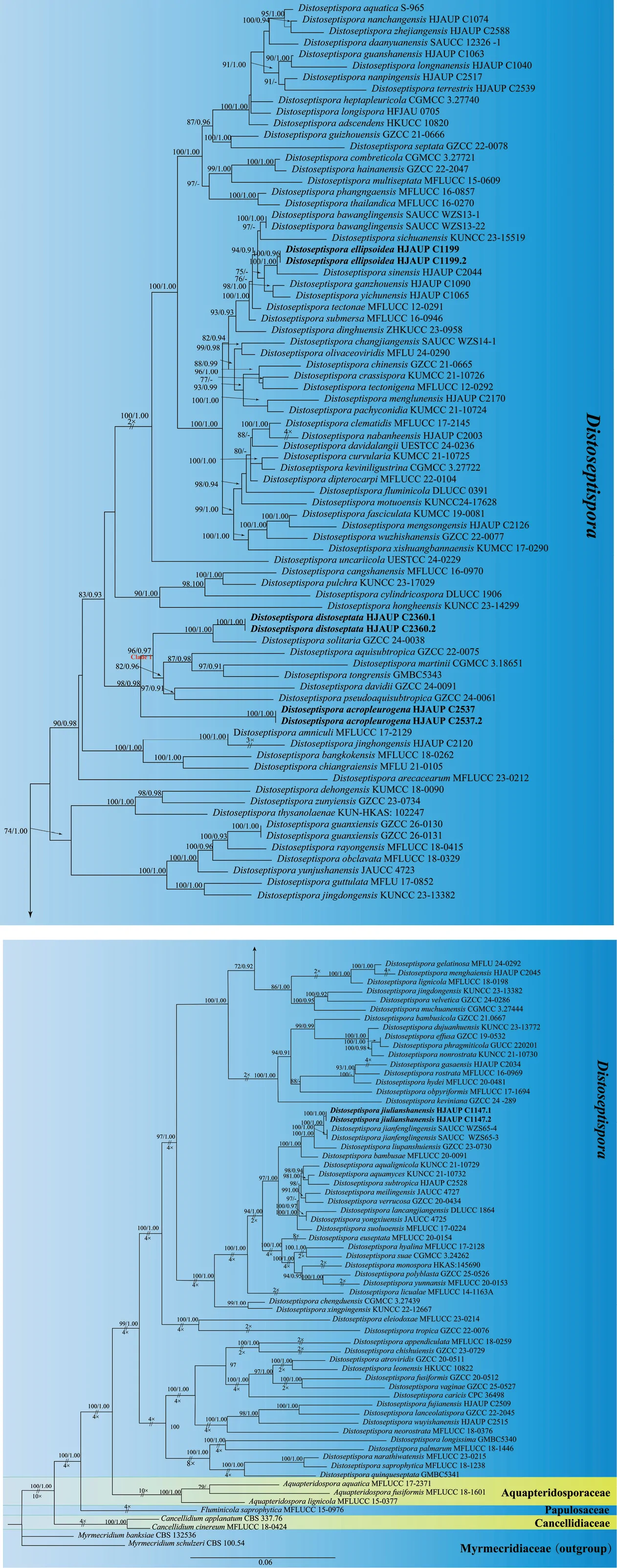
Maximum-likelihood majority-rule consensus tree for *Distoseptisporaceae* and related families using ITS, LSU, *RPB2*, and *TEF1* sequence data. Maximum-likelihood bootstrap support values greater than 75% and Bayesian posterior probabilities greater than 0.90 are shown near the nodes. The tree is rooted with *Myrmecridium schulzeri* (CBS 100.54) and *M. banksiae* (CBS 132536). Strains from the current study are in bold. Some branches were shortened according to the indicated multipliers, and these are indicated by the symbol (//).

Based on the combined sequences of ITS, LSU, *RPB2*, and *TEF1*, eight strains from the present study were analyzed for their phylogenetic relationships within the family *Distoseptisporaceae* and related families (*Aquapteridosporaceae*, *Cancellidiaceae*, and *Papulosaceae*), using 141 strains representing 135 species (Table 1). *Myrmecridium banksiae* (CBS 132536) and *M. schulzeri* (CBS 100.54) were used as the outgroup. The combined sequence alignment consisted of 3,647 columns (ITS: 1–692, LSU: 693–1,629, *RPB2*: 1,630–2,704, *TEF1*: 2,705–3,647), including 2,051 distinct patterns, 1,444 parsimony-informative sites, 341 singleton sites, and 1,861 constant sites. Maximum-likelihood (ML) and Bayesian inference (BI) analyses of the combined sequences yielded phylogenetic reconstructions with essentially similar topologies. The best-scoring ML tree (lnL = –53813.334), with ultrafast bootstrap values from ML analyses and posterior probabilities from the MrBayes analysis shown as the first and second values above the nodes, respectively, is presented in Fig. 1. Phylogenetic analyses of the ITS+LSU+*RPB2*+*TEF1* concatenated datasets showed that these eight strains nested within the genus *Distoseptispora*, representing four independent lineages (Fig. 1). *Distoseptispora acropleurogena* (HJAUP C2537 and HJAUP C2537.2) formed an independent lineage basal to Clade 1 with 98% ML/0.98 BI bootstrap support. *Distoseptispora distoseptata* (HJAUP C2360.1 and HJAUP C2360.2) formed a distinct clade sister to *D. solitaria* (GZCC 24-0038) with 100% ML/1.0 BI bootstrap support. *Distoseptispora ellipsoidea* (HJAUP C1199 and HJAUP C1199.2) clustered as sister to *D. sinensis* (HJAUP C2044) with 100% ML/1.0 BI support. *Distoseptispora jiulianshanensis* (HJAUP C1147.1 and HJAUP C1147.1) clustered as a sister taxon to *D. jianfenglingensis* (SAUCC WZS65-3 and SAUCC WZS65-4) with 100% ML/1.0 BI bootstrap support.

### Taxonomy

#### Distoseptispora acropleurogena

Taxon classificationFungiDistoseptisporalesDistoseptisporaceae

M.G. Liao & Jian Ma sp. nov.

DAF6BDD8-DFFA-5F52-8862-5FC835B44181

Index Fungorum: IF905892

Fig. 2

##### Type.

China • Fujian Province, Nanping City, Wuyishan National Nature Reserve, 27°43'N, 117°41'E, on dead branches of an unidentified broadleaf tree, 16 November 2023, M.G. Liao (HJAUP M2537, ***holotype***), ex-type living culture HJAUP C2537 = HJAUP C2537.2.

##### Etymology.

In reference to the acropleurogenous conidia.

##### Description.

Saprobic on dead branches in a terrestrial habitat. Sexual morph: Undetermined. Asexual morph: Colonies on natural substrate effuse, scattered, dark brown to black, hairy. Mycelium partly superficial, partly immersed in the substratum, composed of branched, septate, smooth, pale brown to brown hyphae. Conidiophores macronematous, mononematous, unbranched, solitary, erect, straight or slightly flexuous, cylindrical, smooth, 6–12-septate, brown to dark brown and paler towards the apex, 131–232 × 5.5–7.5 µm (x̄ = 191.3 × 6.4 µm, *n* = 30). Conidiogenous cells monoblastic or polyblastic, integrated, terminal, cylindrical, pale brown to brown, smooth, determinate. Conidial secession schizolytic. Conidia acropleurogenous, solitary, dry, straight or curved, obclavate, rostrate, smooth or verrucose, 6–10-euseptate, pale brown to brown, 53–86 × 7–14 µm (x̄ = 66.2 × 10.8 µm, *n* = 30), tapering to 2–4 µm near the apex, 4–5.5 µm wide at the truncate base.

**Figure 2.**
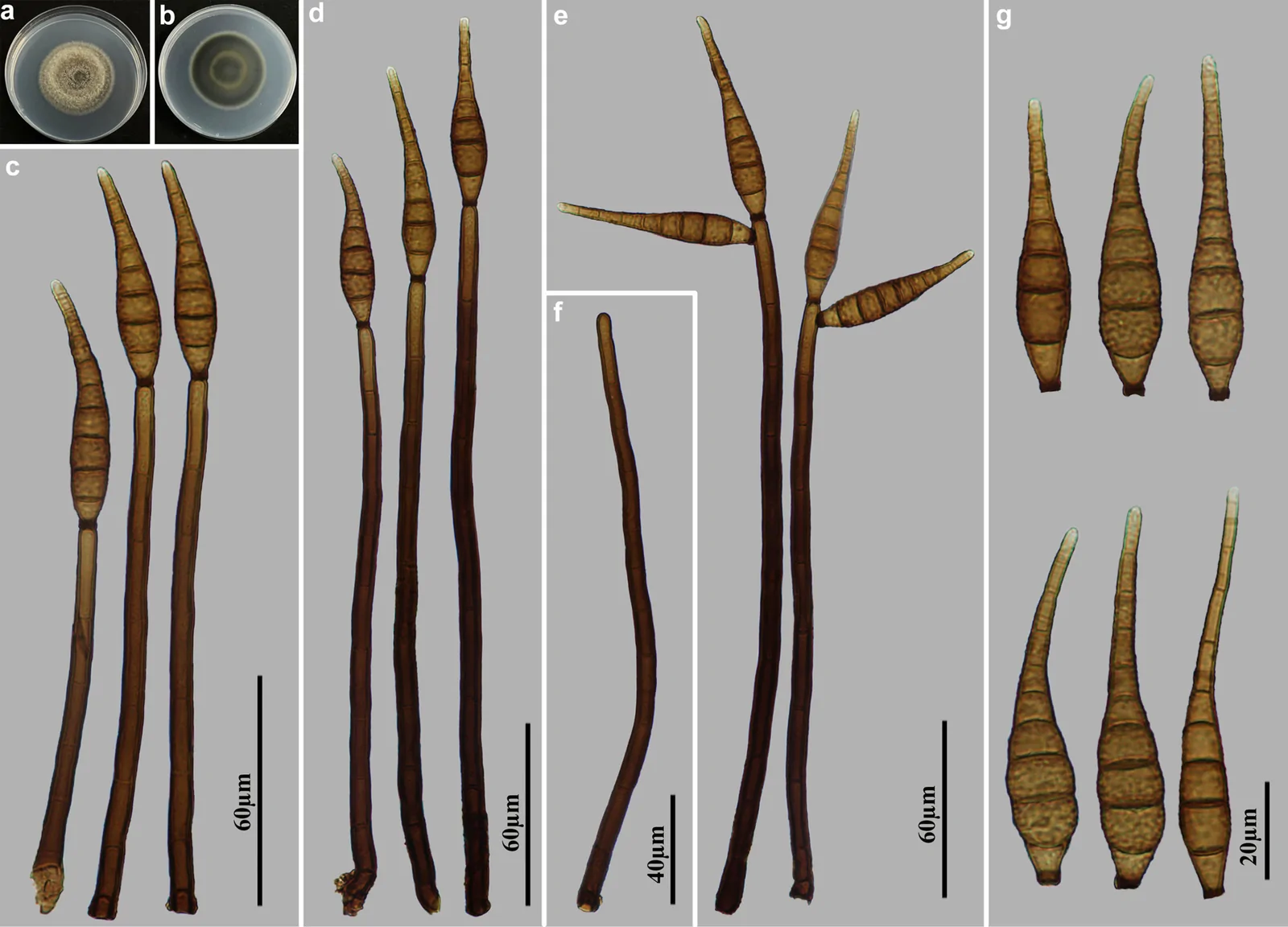
*Distoseptispora acropleurogena* (HJAUP M2537, holotype). **a, b**. Colonies on PDA after 4 weeks (front and reverse); **c–e**. Conidiophores, conidiogenous cells, and conidia; **f**. Conidiophore; **g**. Conidia.

##### Culture characteristics.

Colonies on PDA reaching 62 mm diam. after 4 weeks in an incubator under dark conditions at 25 °C, circular, flattened, with a velvety surface, yellow to pale brown with dense mycelium, margin entire; reverse dark brown in the central part with a pale brown inner ring and an outer pale brown halo.

##### Notes.

Phylogenetic analyses showed that *D. acropleurogena* (HJAUP C2357 and HJAUP C2357.2) belongs to *Distoseptispora* and forms a distinct lineage sister to Clade 1 with 98% ML/0.98 BI bootstrap support. However, *D. acropleurogena* (HJAUP C2357) differs from its closest relatives, *D. davidii* (GZCC 24-0091) by 202 bp nucleotide differences (38/499 in ITS, including 16 gaps; 10/561 in LSU, including 2 gaps; 105/1041 in *RPB2*, including 9 gaps; 49/931 in *TEF1*, no gaps) and *D. pseudoaquisubtropica* (GZCC 24-0061) by 97 bp nucleotide differences (34/496 in ITS, including 13 gaps; 8/558 in LSU, including 1 gap; 55/917 in *TEF1*, no gaps). In morphology, the conidiogenous cells of *D. acropleurogena* are monoblastic or polyblastic, whereas those of *D. davidii* X.Juan Xiao, N.G. Liu & Y.Z. Lu and *D. pseudoaquisubtropica* X.Juan Xiao, N.G. Liu & Y.Z. Lu (Xiao et al. 2025) are monoblastic. Moreover, the conidia of *D. acropleurogena* are acropleurogenous, smooth or verrucose, and euseptate, whereas those of *D. davidii* and *D. pseudoaquisubtropica* are acrogenous, smooth, and distoseptate. In addition, *D. acropleurogena* differs from *D. davidii* by having longer conidiophores (131–232 µm vs. 64–104 µm) and smaller conidia (53–86 × 7–14 µm vs. 93–177 × 12–17 µm), and from *D. pseudoaquisubtropica* by having longer and narrower conidiophores (131–232 × 5.5–7.5 µm vs. 31–50 × 7.5–10 µm) and smaller conidia (53–86 × 7–14 µm vs. 284–423 × 12–19 µm). Based on morphological and phylogenetic analyses, *D. acropleurogena* is described as a new species.

#### Distoseptispora distoseptata

Taxon classificationFungiDistoseptisporalesDistoseptisporaceae

F.H. Guo & Jian Ma sp. nov.

3F11FC45-4F37-52C6-8C4C-9BCFE9A71968

Index Fungorum: IF905893

Fig. 3

##### Type.

China • Yunnan Province, Jinghong City, Nabanhe National Nature Reserve, 22°04'N, 100°32'E, on dead branches of an unidentified broadleaf tree, 12 July 2022, J.W. Liu (HJAUP M2360, ***holotype***), ex-type living culture HJAUP C2360.1 = 2360.2.

**Figure 3.**
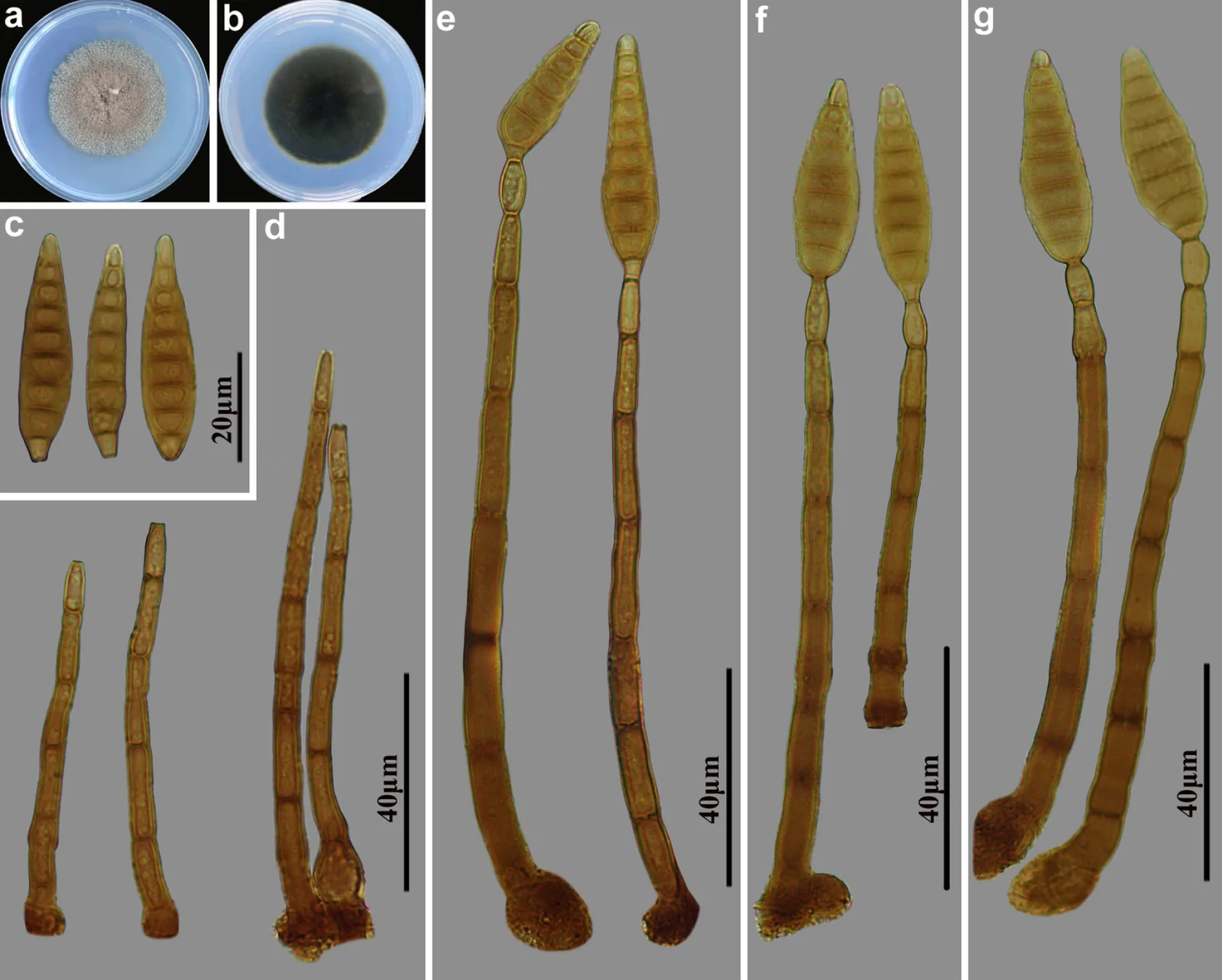
*Distoseptispora distoseptata* (HJAUP M2360, holotype). **a, b**. Colonies on PDA after 4 weeks (front and reverse); **c**. Conidia; **d**. Conidiophores; **e–g**. Conidiophores, conidiogenous cells, and conidia.

##### Etymology.

In reference to the distoseptate conidia of the species.

##### Description.

Saprobic on dead branches in a terrestrial habitat. Sexual morph: Undetermined. Asexual morph: Colonies on natural substrate effuse, scattered, brown to dark brown, hairy. Mycelium partly superficial, partly immersed in the substratum, composed of branched, septate, smooth, pale brown to brown hyphae. Conidiophores macronematous, solitary, unbranched, erect or slightly flexuous, cylindrical, base sometimes slightly swollen, brown, paler towards apex, smooth-walled, 4–7-septate, 74–134 × 5–8.2 µm (x̄ = 106 × 6.7 µm, *n* = 30). Conidiogenous cells integrated, terminal, monoblastic, cylindrical, pale brown, smooth-walled, determinate, apex truncate. Conidial secession schizolytic. Conidia acrogenous, solitary, dry, obclavate or fusiform, straight or slightly curved, brown, paler towards the apex, base truncate, apex obtuse, smooth, mostly 8-distoseptate when mature, 32–46 × 7.6–14.4 µm (x̄ = 38.18 × 11.81 µm, *n* = 30), tapering to 2.4–4.7 µm near the apex, 2.2–3.5 µm wide at the truncate base.

##### Culture characteristics.

Colonies on PDA reaching 58–62 mm diam. after 4 weeks in an incubator under dark conditions at 25 °C, circular, surface velvety, with grayish brown and denser mycelium at the center, becoming dark brown at the margin with an obvious boundary, reverse dark brown to black, paler toward the boundary.

##### Notes.

Phylogenetic analyses showed that *D. distoseptata* (HJAUP C2360.1 and HJAUP C2360.2) clusters with *D. solitaria* (GZCC 24-0038) with 100% ML/1.0 BI bootstrap support. BLASTn analysis revealed that the nucleotide comparison between *D. distoseptata* (HJAUP C2360.1) and *D. solitaria* (GZCC 24-0038) showed 27/559 (4.8% difference, including 11 gaps) in ITS and 3/881 (0.3% difference, no gaps) in LSU; no data are available for *RPB2* or *TEF1* of *D. solitaria* (GZCC 24-0038) in GenBank. In morphology, the conidia of *D. distoseptata* are predominantly 8-distoseptate when mature, whereas those of *D. solitaria* X.Juan Xiao, N.G. Liu & Y.Z. Lu (Xiao et al. 2025) are mostly 9-distoseptate when mature. In addition, *D. distoseptata* differs from *D. solitaria* in its smaller conidiophores (74–134 × 5.0–8.2 µm vs. 91–188 × 5–9 µm) and shorter and wider conidia (32–46 × 7.6–14.4 µm vs. 32–54 × 8–11 µm). Based on morphological and phylogenetic differences and the criteria established by Jeewon and Hyde (2016) and Chethana et al. (2021), *D. distoseptata* is described as a new species.

#### Distoseptispora ellipsoidea

Taxon classificationFungiDistoseptisporalesDistoseptisporaceae

M.G. Liao & Jian Ma sp. nov.

2F41F072-0B54-5DD9-80B9-397832642F67

Index Fungorum: IF905894

Fig. 4

##### Type.

China • Jiangxi Province, Yichun City, Guanshan National Nature Reserve, 28°32'N, 114°33'E, on dead branches of an unidentified broadleaf tree, 25 June 2021, Y.F. Hu (HJAUP M1199, ***holotype***), ex-type living culture HJAUP C1199 = HJAUP C1199.2.

**Figure 4.**
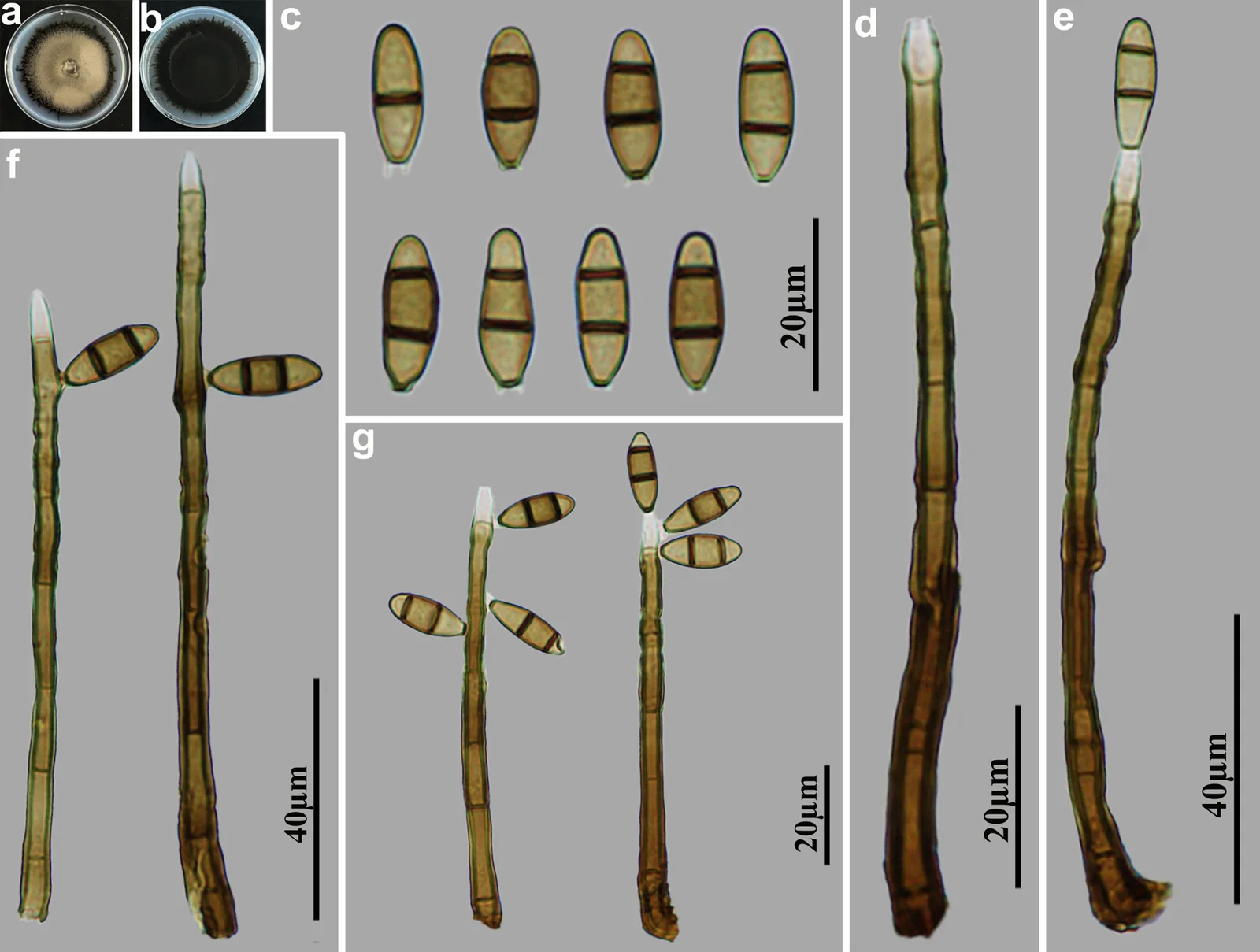
*Distoseptispora ellipsoidea* (HJAUP M1199, holotype). **a, b**. Colonies on PDA after 4 weeks (front and reverse); **c**. Conidia; **d**. Conidiophore; **e–g**. Conidiophore, conidiogenous cells, and conidia. Note that in **f** and **g**, the mature conidium is pushed sideways following the successive percurrent extension of the conidiogenous cells.

##### Etymology.

In reference to the ellipsoidal conidia.

##### Description.

Saprobic on dead branches in a terrestrial habitat. Sexual morph: Undetermined. Asexual morph: Colonies effuse, scattered, smooth, hairy, dark brown to black. Mycelium immersed and superficial, composed of septate, branched, pale brown to brown hyphae. Conidiophores macronematous, mononematous, solitary, unbranched, smooth, cylindrical, straight, 4–9-septate, brown, 82.5–125.8 × 3.1–6.2 µm (x̄ = 104.1 × 4.7 µm, *n* = 30). Conidiogenous cells monoblastic, terminal, integrated, cylindrical, smooth, brown, percurrent, attenuated into narrow and truncate at the apex. Conidial secession rhexolytic. Conidia acrogenous, solitary, ellipsoidal, (1–)2-euseptate, smooth, brown, 7.8–18.7 × 2.2–6.9 µm (x̄ = 13.4 × 4.6 µm, *n* = 30), apex rounded, base truncate, with a distinct basal frill derived from the distal end of the conidiogenous cell.

##### Culture characteristics.

Colonies on PDA reaching 81 mm diam. after 4 weeks in an incubator under dark conditions at 25 °C, circular, surface velvety, with white to pale brown and denser mycelium, becoming black at the margin, reverse black.

##### Notes.

Phylogenetic analyses showed that *D. ellipsoidea* (HJAUP C1199 and HJAUP C1199.2) clusters with *D. sinensis* (HJAUP C2044) with 100% ML/1.0 BI bootstrap support. BLASTn analysis revealed 10 bp nucleotide differences between *D. ellipsoidea* (HJAUP C1199) and *D. sinensis* (HJAUP C2044) (5/606 in ITS, including 3 gaps; 1/541 in LSU, no gaps; 4/900 in *TEF1*, no gaps). In morphology, *D. ellipsoidea* has ellipsoidal conidia with few eusepta (1–2), whereas *D. sinensis* Jing W. Liu, X.G. Zhang & Jian Ma (Liu et al. 2023) has obclavate conidia with numerous distosepta (10–25). Moreover, the mode of conidial secession in *D. ellipsoidea* is rhexolytic, whereas that of *D. sinensis* is schizolytic. In addition, *D. ellipsoidea* differs from *D. sinensis* in its percurrently extending, longer conidiophores (82.5–125.8 µm vs. 23.5–56.5 µm) and smaller conidia (7.8–18.7 × 2.2–6.9 µm vs. 40–107 × 10–12 µm). Based on distinct morphological characters and molecular phylogeny, *D. ellipsoidea* is described as a new species.

#### Distoseptispora jiulianshanensis

Taxon classificationFungiDistoseptisporalesDistoseptisporaceae

F.H. Guo & Jian Ma sp. nov.

49F1ACB8-A283-5589-A268-2123D6076035

Index Fungorum: IF905895

Fig. 5

##### Type.

China • Jiangxi Province, Ganzhou City, Jiulianshan National Nature Reserve, 24°29'N, 114°22'E, on dead branches of an unidentified broadleaf tree, 18 July 2022, Y.F. Hu (HJAUP M1147, ***holotype***), ex-type living culture HJAUP C1147.1 = 1147.2.

**Figure 5.**
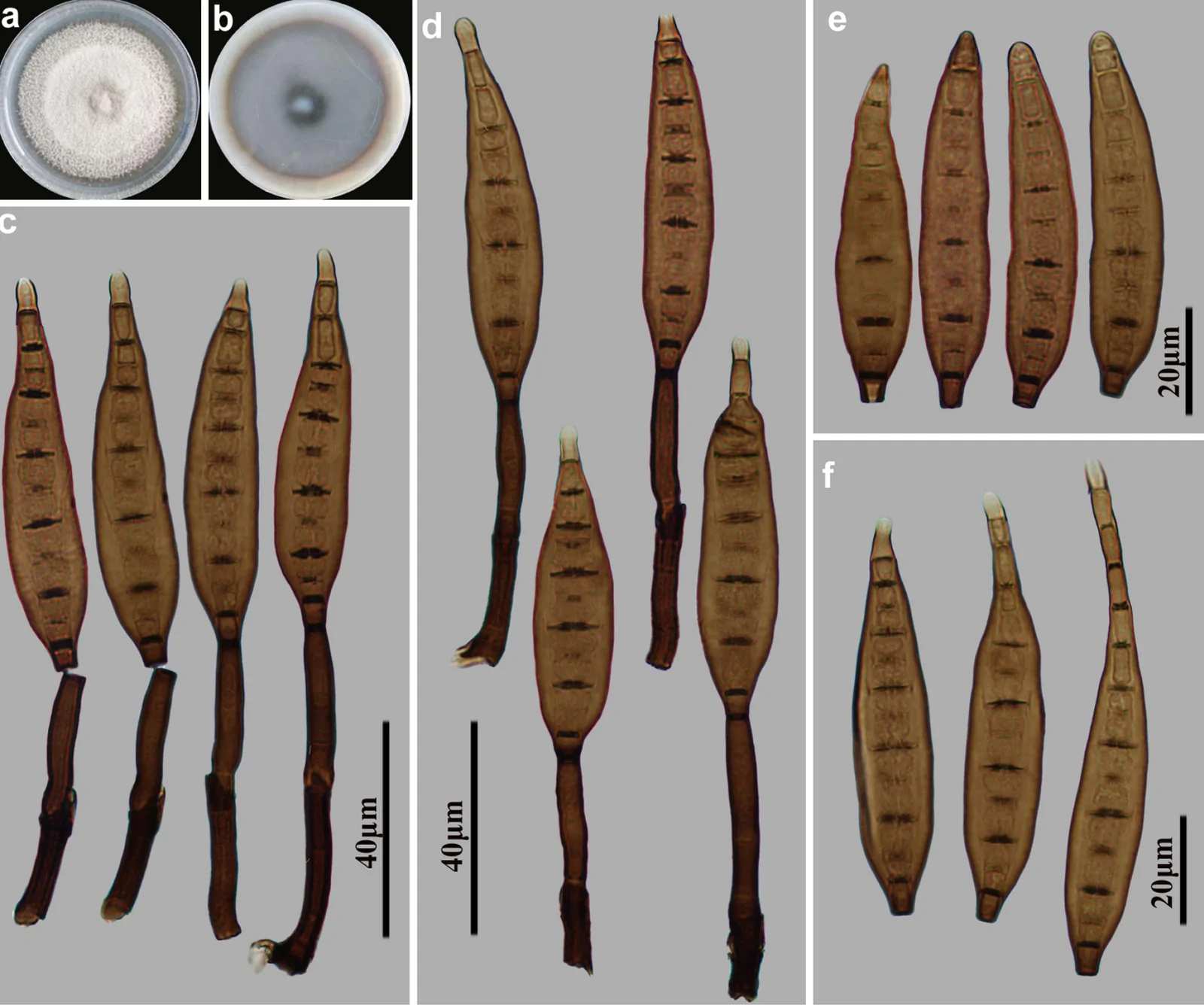
*Distoseptispora jiulianshanensis* (HJAUP M1147, holotype). **a, b**. Colonies on PDA after 4 weeks (front and reverse); **c, d**. Conidiophore, conidiogenous cells, and conidia; **e, f**. Conidia.

##### Etymology.

In reference to the type locality, Jiulianshan National Nature Reserve.

##### Description.

Saprobic on dead branches in a terrestrial habitat. Sexual morph: Undetermined. Asexual morph: Colonies on natural substrate effuse, scattered, dark brown to black, hairy. Mycelium partly superficial, partly immersed in the substratum, composed of branched, septate, smooth, pale brown to brown hyphae. Conidiophores macronematous, mononematous, solitary or in groups of 2–3, unbranched, erect, straight or slightly flexuous, cylindrical, base slightly swollen, dark brown to blackish brown, paler towards the apex, smooth-walled, 4–8-septate, 49–68 × 4.4–5.9 µm (x̄ = 56.89 × 5.0 µm, *n* = 30), determinate or with one cylindrical percurrent extension. Conidiogenous cells integrated, terminal, monoblastic, cylindrical, pale brown to brown, smooth-walled, with a flat apex. Conidial secession schizolytic. Conidia acrogenous, solitary, dry, obclavate to fusiform, straight or slightly curved, brown to dark brown, paler towards the apex, base truncate, apex gradually tapering into a short rostrum, 9–12-distoseptate and most 11-distoseptate, smooth, not constricted at the septa, 58–98 × 13–17 µm (x̄ = 74.22 × 14.67 µm, *n* = 30), tapering to a hyaline to pale brown rostrum near the apex, 3.5–4.8 µm wide at the truncate base.

##### Culture characteristics.

Colonies on PDA reaching 75–79 mm diam. after 4 weeks in an incubator under dark conditions at 25 °C, circular, surface velvety, with pale brown and denser mycelium at the center, becoming grayish brown at the margin, reverse dark brown to black at the center, paler toward the boundary.

##### Notes.

Phylogenetic analyses showed that *D. jiulianshanensis* (HJAUP C1147.1 and HJAUP C1147.2) clusters with *D. jianfenglingensis* (SAUCC WZS65-3 and SAUCC WZS65-4) with 100% ML/1.0 BI bootstrap support. BLASTn analysis revealed 7 bp nucleotide differences between *D. jiulianshanensis* (HJAUP C1147.1) and *D. jianfenglingensis* (SAUCC WZS65-3) (3/591 in ITS, including 1 gap; 2/1094 in *RPB2*, including 1 gap; 2/923 in *TEF1*, including 1 gap). In morphology, *D. jiulianshanensis* has smooth-walled conidia with 9–12 (usually 11) distosepta, whereas *D. jianfenglingensis* W.W. Liu, C.Z. Yin, X.G. Zhang & S. Wang (Liu et al. 2025) has rough-walled conidia with 5–10 (usually 9) distosepta. In addition, *D. jiulianshanensis* differs from *D. jianfenglingensis* in its shorter conidiophores (49–68 µm vs. 66.5–164.2 µm) and larger conidia (58–98 × 13–17 µm vs. 43.3–76.1 × 5.1–7.5 µm), and further differs from *D. jianfenglingensis* in its conidiophores, which usually have a single cylindrical percurrent extension that appears to represent regenerative growth. Based on morphological and phylogenetic differences, *D. jiulianshanensis* is described as a new species.

## Discussion

In this study, four new species of *Distoseptispora* from terrestrial habitats in southern China, namely *D. acropleurogena*, *D. distoseptata*, *D. ellipsoidea*, and *D. jiulianshanensis*, were described based on morphological comparisons and multilocus phylogenetic analyses of combined ITS, LSU, *RPB2*, and *TEF1* sequence data (Su et al. 2016; Hu et al. 2023). These findings enrich the species diversity of *Distoseptispora* in southern China and extend its documented distribution into Fujian, Jiangxi, and Yunnan provinces. The four new species were collected from dead branches of unidentified broadleaf trees in terrestrial forests, consistent with the known saprobic ecology of the genus (Song et al. 2020; Li et al. 2021). The results suggest that the diversity of *sporidesmium*-like fungi in southern China still requires further exploration, which will contribute to a better understanding of these fungi and clarify their taxonomic status through phylogenetic analyses.

Recent studies indicate that the use of ITS, LSU, *RPB2*, and *TEF1* provides greater resolution in delineating *Distoseptispora* species (Liu et al. 2025; Xiao et al. 2025). Accordingly, phylogenetic analyses were also conducted using ITS, LSU, *RPB2*, and *TEF1* sequences, and the eight newly obtained strains nested within the genus *Distoseptispora* and formed distinct clades with high phylogenetic support. However, cases of discordance between molecular and morphological data still exist. For instance, *D. ellipsoidea* and *D. sinensis* are sister species in phylogenetic analyses, and their sequence divergence is surprisingly low (0.8% in ITS, 0.2% in LSU, and 0.4% in *TEF1*). However, both species exhibit striking morphological differences. The former produces ellipsoidal, 7.8–18.7 × 2.2–6.9 µm, (1–)2-euseptate conidia seceding rhexolytically from percurrently extending conidiophores, whereas the latter has obclavate, 40–107(–137) × 10–12 µm, 10–25-distoseptate conidia seceding schizolytically from determinate conidiophores. Similarly, *D. jiulianshanensis* also exhibits very low phylogenetic divergence from *D. jianfenglingensis* (0.5% in ITS, 0.2% in *RPB2*, and 0.2% in *TEF1*). However, both species can be clearly distinguished by conidial shape, size, septation, and ornamentation. Such inconsistencies highlight that relying solely on phylogenetics to infer relationships among closely related species may require additional gene loci for combined analysis and that morphological evidence remains indispensable for species identification, especially when molecular signals are weak or conflicting.

At present, studies on *Distoseptispora* have mainly focused on its species diversity and taxonomic classification. Over the past few years, the number of species has steadily increased and now stands at 131, including the four species described in this study. Of these, only two species, *D. hyalina* and *D. licualae*, have been described as sexual morphs based on molecular DNA data (Yang et al. 2021; Konta et al. 2023), but there are no reports of a connection between *Distoseptispora* species and their teleomorphs. Most *Distoseptispora* species are considered to be saprobes of chiefly woody hosts in terrestrial and freshwater habitats (Liu et al. 2023; Liao et al. 2025; Liu et al. 2025; Xiao et al. 2025; Index Fungorum 2026) and have the ability to decompose lignocellulose in wood (Wong et al. 1998; Hyde et al. 2016; Zhai et al. 2022), but little attention has been accorded to their ecosystem function in decomposition and nutrient recycling. Thus, studying their biology and ecological roles using a metagenomic approach or enzymatic technology is needed and may help provide insights into their functional significance.

## Supplementary Material

XML Treatment for Distoseptispora acropleurogena

XML Treatment for Distoseptispora distoseptata

XML Treatment for Distoseptispora ellipsoidea

XML Treatment for Distoseptispora jiulianshanensis

## Additional information

### Artificial Intelligence (AI) use

The authors accept full responsibility for the content of the manuscript, including the disclosure of any use of AI.

No AI tools were used in the preparation of this manuscript.

### Author ORCIDs

F.-H. Guo https://orcid.org/0009-0003-1346-4525

Z.-H. Wang https://orcid.org/0009-0006-5224-8325

M.-G. Liao https://orcid.org/0009-0001-9537-1773

L.-H. Zhang https://orcid.org/0000-0001-7427-3720

Z.-H. Xu https://orcid.org/0009-0008-2641-7783

W.-G. Kuang https://orcid.org/0000-0002-7617-3085

J. Ma https://orcid.org/0000-0001-9783-1860

### Data availability

All of the data that support the findings of this study are available in the main text or Supplementary Information.

## Supplementary materials

Supplementary material 1 The concatenated ITS, LSU, RPB2 and TEF1 sequences Data type fas https://binary.pensoft.net/file/1767469This dataset is made available under the Open Database License (http://opendatacommons.org/licenses/odbl/1.0/). The Open Database License (ODbL) is a license agreement intended to allow users to freely share, modify, and use this Dataset while maintaining this same freedom for others, provided that the original source and author(s) are credited. Fang-Hua Guo, Zhi-Hao Wang, Ming-Gen Liao, Lian-Hu Zhang, Ru-Qiang Cui, Zhao-Huan Xu, Wei-Gang Kuang, Jian Ma
